# Lower hand grip strength in older adults with non-alcoholic fatty liver disease: a nationwide population-based study

**DOI:** 10.18632/aging.102068

**Published:** 2019-07-07

**Authors:** Beom-Jun Kim, Seong Hee Ahn, Seung Hun Lee, Seongbin Hong, Mark W. Hamrick, Carlos M. Isales, Jung-Min Koh

**Affiliations:** 1 Division of Endocrinology and Metabolism, Asan Medical Center, University of Ulsan College of Medicine, Seoul, Republic of Korea; 2 Division of Endocrinology and Metabolism, Inha University Hospital, Inha University School of Medicine, Incheon, Republic of Korea; 3 Department of Cellular Biology and Anatomy, Medical College of Georgia, Augusta University, Augusta, GA 30912, USA; 4 Department of Medicine, Medical College of Georgia, Augusta University, Augusta, GA 30912, USA

**Keywords:** non-alcoholic fatty liver disease, hand grip strength, sarcopenia, muscle strength

## Abstract

Although both liver and muscle are metabolically active endocrine organs, and non-alcoholic fatty liver disease (NAFLD) and sarcopenia may share common pathogenic determinants, there have been few clinical studies of the relationship between NAFLD and muscle strength, especially in the elderly. We conducted a nationally representative population-based, cross-sectional study using data from the Korea National Health and Nutrition Examination Survey, which involved 1,897 men aged ≥50 years and 2,206 postmenopausal women. NAFLD was defined using the hepatic steatosis index (HSI) and low muscle strength was defined using the Korea-specific cut-off point of hand grip strength (HGS). Men and women with NAFLD had 7.3% and 7.9% lower HGS than controls, respectively. The odds ratios for low muscle strength in the presence of NAFLD were 2.51 in men and 2.34 in women. HSI inversely correlated with HGS in both men and women. Consistently, compared with men and women in the lowest HSI quartile, those in the highest quartile had 7.6% and 12.4% lower HGS, respectively, and were 5.63- and 3.58-times more likely to have low muscle strength, respectively. These results provide the first clinical evidence that NAFLD can be associated with muscular impairment in older adults, as demonstrated by lower muscle strength.

## INTRODUCTION

Hand grip strength (HGS) is a measure of the maximum static force that a hand can apply around a dynamometer. It is considered to be a readily feasible and convenient indicator of overall muscle strength with good test-retest reliability and responsiveness [1, 2]. Low HGS has been consistently linked to not only a range of health impairments, such as comorbidities, falls, disability, and poor quality of life, but also higher all-cause mortality [3–7]. Given its predictive validity and simplicity, the intriguing suggestion has been made that grip strength might have potential as a screening tool in middle-aged and older adults [5, 8]. Importantly, muscle strength is known to be better than muscle mass in predicting adverse outcomes [3, 9, 10], and therefore the recently revised guidelines issued by the European Working Group on Sarcopenia in Older People (EWGSOP) focused on low muscle strength as the primary measure of sarcopenia [11]. Thus, the importance of muscle strength is increasingly recognized in research and clinical practice to improve human muscle health.

Non-alcoholic fatty liver disease (NAFLD) is defined as the accumulation of excessive fat in the liver of individuals in the absence of significant alcohol consumption. NAFLD encompasses a wide spectrum of disorders, ranging from simple steatosis to non-alcoholic steatohepatitis (NASH), which can progress to fibrosis or even cirrhosis [12]. The significance of NAFLD for public health has been the subject of substantial recent attention because NAFLD is predictive of cardiovascular diseases and greater mortality [13–15], and because its prevalence is increasing worldwide as a result of the obesity epidemic [16, 17]. Interestingly, both liver and muscle are active endocrine organs that can secrete substances exerting metabolic effects [18–20], and NAFLD and sarcopenia may share common pathogenic mechanisms, such as insulin resistance and chronic inflammation [21, 22]. Indeed, several epidemiologic studies [23–27] and review papers [28–30] have suggested a possible link between NAFLD and sarcopenia. However, these studies have focused on the significance of muscle mass, which can only partially explain muscle strength, and clinical studies aimed at determining the nature of any relationship between NAFLD and muscle strength have been few in number, especially in the elderly, who represent the population that is most vulnerable to sarcopenia and related outcomes. Thus, the aim of the present study was to evaluate the relationship between NAFLD status and HGS using a cohort that was representative of the general Korean population.

## RESULTS

Table 1 shows the clinical characteristics of the participants according to sex and NAFLD status. Among 1,897 men aged ≥50 years and 2,206 postmenopausal women, 332 (17.5%) male and 562 (25.5%) female subjects had NAFLD. Among the men, the participants with NAFLD were younger and had higher weight, body mass index (BMI), systolic and diastolic blood pressure (BP), serum triglycerides, fasting plasma glucose, glycated hemoglobin A1c (HbA1c), serum aspartate aminotransferase (AST), alanine aminotransferase (ALT), and hepatic steatosis index (HSI) than those without NAFLD, but there were no differences in height, smoking habit, resistance exercise, or serum total cholesterol between the two groups. Among the women, the participants with NAFLD had higher weight, BMI, systolic and diastolic BP, serum triglycerides, fasting plasma glucose, HbA1c, serum AST, ALT, and HSI, and lower height and serum total cholesterol than those without NAFLD. However, there were no differences in age, smoking habit, or resistance exercise between the two groups. Crude analyses showed that men with NAFLD had significantly higher HGS than those without, but the difference in HGS according to NAFLD status was not identified in women.

**Table 1:** Baseline characteristics of the study participants, categorized according to the presence or absence of NAFLD.

**Variable**	**Men (*n* = 1,897)**			**Women (*n* = 2,206)**		
	**Without NAFLD (*n* = 1,565)**	**With NAFLD (*n* = 332)**	***P***	**Without NAFLD (*n* = 1,644)**	**With NAFLD (*n* = 562)**	***P***
Age (years)	**61.9 ± 8.7**	**59.5 ± 7.8**	**< 0.001**	61.8 ± 9.1	62.0 ± 9.0	0.623
Weight (kg)	**65.3 ± 8.3**	**78.0 ± 8.1**	**< 0.001**	**54.8 ± 6.4**	**65.6 ± 8.1**	**<0.001**
Height (cm)	167.3 ± 6.1	168.0 ± 6.5	0.123	**154.7 ± 5.9**	**154.0 ± 5.5**	**0.035**
Body mass index (kg/m^2^)	**23.3 ± 2.4**	**27.6 ± 2.4**	**<0.001**	**22.9 ± 2.3**	**27.6 ± 2.8**	**<0.001**
Systolic BP (mmHg)	**122.7 ± 15.7**	**125.3 ± 15.3**	**0.017**	**121.5 ± 17.7**	**127.0 ± 17.3**	**<0.001**
Diastolic BP (mmHg)	**76.4 ± 10.0**	**80.3 ± 10.6**	**<0.001**	**74.1 ± 9.7**	**76.5 ± 9.9**	**<0.001**
Smoking habit (%)			0.215			0.753
Never	17.8	19.4		94.2	93.2	
Past	50.9	55.3		2.8	3.4	
Current	31.3	25.3		3.0	3.4	
Resistance exercise (%)			0.068			0.068
None	66.4	73.0		84.0	88.2	
Intermittent	14.2	9.6		9.2	5.9	
Regular	19.4	17.3		6.8	5.9	
Serum total cholesterol (mg/dL)	187.8 ± 34.6	191.5 ± 35.9	0.138	**200.5 ± 35.8**	**195.9 ± 38.2**	**0.039**
Serum triglycerides (mg/dL)	**154.0 ± 121.7**	**196.8 ± 114.8**	**<0.001**	**125.2 ± 79.9**	**153.3 ± 75.5**	**<0.001**
Fasting plasma glucose (mg/dL)	**104.8 ± 24.9**	**116.2 ± 28.5**	**<0.001**	**99.4 ± 21.1**	**112.9 ± 30.1**	**<0.001**
HbA1c (%)	**5.85 ± 0.83**	**6.30 ± 1.10**	**<0.001**	**5.79 ± 0.70**	**6.25 ± 0.93**	**<0.001**
Serum AST (IU/L)	**23.8 ± 10.3**	**27.8 ± 13.0**	**<0.001**	**22.5 ± 8.2**	**25.3 ± 13.1**	**<0.001**
Serum ALT (IU/L)	**20.3 ± 9.8**	**38.9 ± 3.1**	**<0.001**	**17.2 ± 7.9**	**28.3 ± 17.4**	**<0.001**
Hepatic steatosis index	**30.4 ± 3.3**	**38.9 ± 3.1**	**<0.001**	**31.1 ± 2.8**	**39.0 ± 2.8**	**<0.001**
Hand grip strength (kg force)	**36.8 ± 7.3**	**38.1 ± 6.9**	**0.009**	22.7 ± 4.9	22.8 ± 4.9	0.715

Values are presented as mean ± standard deviation, unless otherwise specified. **Bold** numbers indicate statistically significant values. NAFLD, non-alcoholic fatty liver disease; BP, blood pressure; HbA1c, glycated hemoglobin A1c; AST, aspartate aminotransferase; ALT, alanine aminotransferase.

**Figure 1:**
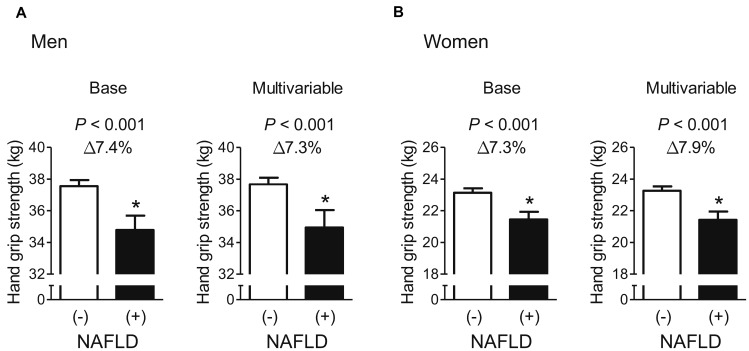
**Difference in hand grip strength between participants with and without NAFLD.** (**A**) Men and (**B**) women. Values are presented as the estimated mean with 95% confidence interval, after adjustment for confounding factors. Delta (Δ) indicates a difference in hand grip strength from controls. ^*^Statistically significant difference from controls. Base model: adjustment for age and weight. Multivariable model: adjustment for age, weight, systolic blood pressure, smoking habit, resistance exercise, total cholesterol, triglycerides, glycated hemoglobin A1c, and alanine aminotransferase. NAFLD, non-alcoholic fatty liver disease.

**Figure 2:**
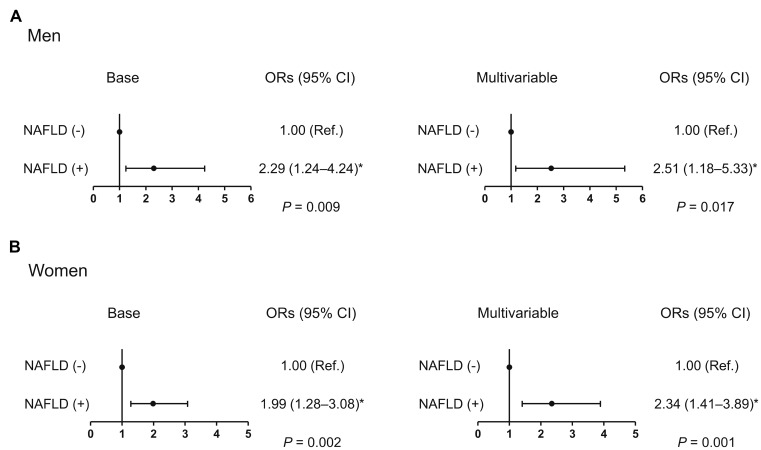
**Odds ratio for low muscle strength in the presence of NAFLD.** (**A**) Men and (**B**) women. ^*^Statistically significant difference from controls. Base model: adjustment for age and weight. Multivariable model: adjustment for age, weight, systolic blood pressure, smoking habit, resistance exercise, total cholesterol, triglycerides, glycated hemoglobin A1c, and alanine aminotransferase. NAFLD, non-alcoholic fatty liver disease; OR, odds ratio; CI, confidence interval.

After adjustment for age and weight, men and women with NAFLD had 7.4% and 7.3% lower HGS, respectively, than those without (Figure 1), and the statistically significant differences in HGS between controls and cases of both sexes persisted, even after systolic BP, smoking, resistance exercise, total cholesterol, triglycerides, HbA1c, and AST were also taken into account. Furthermore, multiple logistic regression analyses revealed that the odds of having low muscle strength were 2.51- and 2.34-fold higher, respectively, in men and women with NAFLD than in controls, in the final adjusted model (Figure 2).

Multiple linear regression analyses were performed to determine which covariates, including HSI, were independently associated with HGS (Table 2). In both men and women, HGS was inversely associated with age and positively associated with weight and resistance exercise. HGS correlated positively with serum total cholesterol, and negatively with serum triglycerides and HbA1c in men, whereas it was positively associated with ALT in women. Importantly, higher HSI was independently associated with lower HGS in both men and women. In addition, each unit increment in HSI was associated with a multivariate-adjusted odds ratio (OR) of 1.17 in men and 1.11 in women for low muscle strength, after adjustment for all potential confounders (Table 3).

**Table 2:** Determination of the covariates, including hepatic steatosis index, that are independently associated with hand grip strength.

**Independent variable**	**Dependent variable: hand grip strength**		
	**β**	**SE**	***P***
Men			
Age	**−0.360**	**0.019**	**<0.001**
Weight	**0.300**	**0.029**	**<0.001**
Systolic BP	0.012	0.011	0.299
Smoking habit	0.037	0.259	0.886
Resistance exercise	**0.493**	**0.220**	**0.025**
Serum total cholesterol	**0.013**	**0.005**	**0.015**
Serum triglycerides	**−0.003**	**0.001**	**0.033**
HbA1c	**−0.415**	**0.194**	**0.033**
Serum ALT	0.009	0.011	0.410
Hepatic steatosis index	**−0.285**	**0.068**	**<0.001**
Women			
Age	**−0.213**	**0.015**	**<0.001**
Weight	**0.239**	**0.023**	**<0.001**
Systolic BP	0.009	0.006	0.189
Smoking habit	−0.494	0.294	0.093
Resistance exercise	**0.450**	**0.166**	**0.007**
Serum total cholesterol	−0.002	0.003	0.543
Serum triglycerides	−0.001	0.001	0.273
HbA1c	−0.084	0.150	0.577
Serum ALT	**0.034**	**0.010**	**0.001**
Hepatic steatosis index	**−0.306**	**0.049**	**<0.001**

The Enter method was applied to this model, using hand grip strength as a dependent variable, and with age, weight, systolic blood pressure, smoking habit, resistance exercise, total cholesterol, triglycerides, HbA1c, ALT, and hepatic steatosis index as independent variables, simultaneously. Values were adjusted for all the other variables in the table. **Bold** numbers indicate statistically significant values. β, regression coefficient; SE, standard error; BP, blood pressure; HbA1c, glycated hemoglobin A1c; ALT, alanine aminotransferase.

**Table 3:** Odds ratios for low muscle strength per unit increase in hepatic steatosis index.

**Men: low muscle strength (HGS < 28.9 kg force)**			**Women: low muscle strength (HGS < 16.8 kg force)**		
**Adjustment**	**ORs (95% CI)**	***P***	**Adjustment**	**ORs (95% CI)**	***P***
Base	**1.11 (1.05–1.17)**	**<0.001**	Base	**1.07 (1.02–1.13)**	**0.010**
Multivariable	**1.17 (1.07–1.28)**	**0.001**	Multivariable	**1.11 (1.02–1.20)**	**0.012**

**Bold** numbers indicate statistically significant values. Base model: adjustment for age and weight. Multivariable model: adjustment for age, weight, systolic blood pressure, smoking habit, resistance exercise, total cholesterol, triglycerides, glycated hemoglobin A1c, and alanine aminotransferase. HGS, hand grip strength; CI, confidence interval.

**Figure 3:**
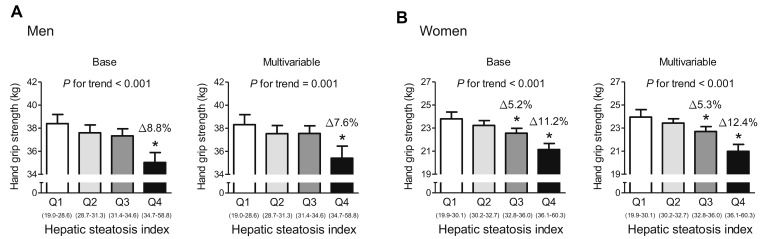
**Hand grip strength, categorized according to hepatic steatosis index quartile.** (**A**) Men and (**B**) women. Values are presented as the estimated mean and 95% confidence interval, after adjustment for confounding factors. Delta (Δ) indicates a difference in hand grip strength from the lowest quartile (Q1). ^*^Statistically significant difference from Q1. Base model: adjustment for age and weight. Multivariable model: adjustment for age, weight, systolic blood pressure, smoking habit, resistance exercise, total cholesterol, triglycerides, glycated hemoglobin A1c, and alanine aminotransferase. NAFLD, non-alcoholic fatty liver disease.

**Figure 4:**
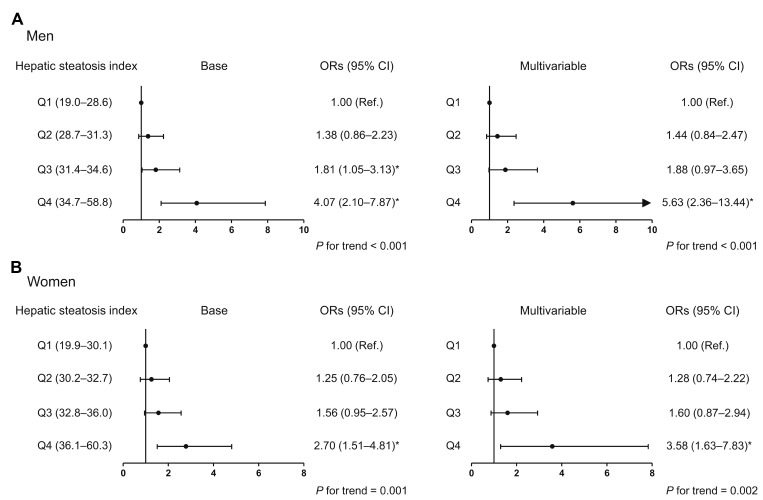
**Odds ratio for low muscle strength, according to hepatic steatosis index quartile.** (**A**) Men and (**B**) women. ^*^Statistically significant difference from the lowest quartile (Q1). Base model: adjustment for age and weight. Multivariable model: adjustment for age, weight, systolic blood pressure, smoking habit, resistance exercise, total cholesterol, triglycerides, glycated hemoglobin A1c, and alanine aminotransferase. OR, odds ratio; CI, confidence interval.

To investigate whether the association between HSI and HGS involves a threshold effect, we divided participants of each sex into quartiles according to HSI (Figure 3). In the base and multivariable adjustment modes, men in the highest quartile (HSI ≥ 34.7) had 8.8% and 7.6% lower HGS than those in the lowest quartile (HSI ≤ 28.6), respectively. Compared with women in the lowest quartile (HSI ≤ 30.1), subjects in the third (32.8 ≤ HIS ≤ 36.0) and highest quartile (HSI ≥ 36.1) had 5.3% and 12.4% lower HGS, respectively, after adjustment for all the potential confounders. Consistent with this, in both men and women, the risk of low muscle strength was 5.63- and 3.58-times higher, respectively, for subjects in the highest HSI quartile than for those in the lowest quartile in the multivariable adjustment model (Figure 4).

Of the 4,103 participants, homeostasis model assessment-estimated insulin resistance (HOMA-IR) and high-sensitivity C-reactive protein (hsCRP) values were available for 2,172 subjects. In this subgroup, when we additionally adjusted for HOMA-IR and hsCRP values in the multivariable model, HGS remained lower in men and women with NAFLD than in controls (Supplementary Figure 1), and independent inverse associations between HSI and HGS persisted in both men and women (Supplementary Table 1).

## DISCUSSION

Although sarcopenia has been regarded as an inevitable outcome of aging for a long time, it is now regarded as a disease that should be overcome. Therefore, many researchers have been trying to understand the pathophysiology of sarcopenia and identify the related risk factors. In this nationally representative, population-based study of older adults, we have shown that men and women with NAFLD have markedly lower HGS and are more likely to have low muscle strength than controls. Consistent with this, higher HSI is associated with lower HGS in both sexes. To the best of our knowledge, this is the first report showing that NAFLD may be associated with muscle deterioration, indicated by lower muscle strength, in community-dwelling elderly people.

Low muscle mass in patients with liver cirrhosis is common and considered to be clinically important in terms of both morbidity and mortality. For example, a systematic review and meta-analysis of 20 studies of the impact of muscle loss on outcomes in liver cirrhosis concluded that there is a higher risk of complications, such as infection and poorer survival rate, in patients with sarcopenia than in those without [31]. Others have also shown that low muscle mass in cirrhotic patients is associated with higher mortality and poor prognosis after liver transplantation [32]. Extrapolating from these findings, the relationship between NAFLD, the most common form of chronic liver disease, and sarcopenia has become a hot topic, due to the importance of both diseases in public healthcare systems. Since Hong *et al.* [23] first identified a potential link between low muscle mass and NAFLD, consistent associations have been shown in subsequent clinical studies [24–27]. There is no doubt that these studies, which defined sarcopenia on the basis of muscle mass, have significantly contributed to our knowledge of muscle-liver cross-talk. However, a gain in muscle mass cannot prevent the decline in muscle strength during aging [33] and low muscle mass is not responsible for the inverse relationship between muscle strength and mortality [34]. Therefore, these previous studies that focused on muscle mass, though important, are not sufficient to fully understand the link between muscle status and NAFLD. In this context, our current study might have important implications because it suggests that NAFLD could be directly associated with muscle strength*,* rather than just muscle mass, during aging.

Several mechanisms have been suggested to explain the disturbances in muscle metabolism that are associated with NAFLD. Insulin resistance and chronic inflammation have been the most frequently proposed mechanisms, and both are hypothetically plausible [28, 30]. However, in the present study, the low muscle strength associated with NAFLD persisted after adjustment for HOMA-IR and hsCRP values in the multivariable model, implying that other mechanisms, independent of insulin resistance and chronic inflammation, may be of more significance. First, lower production of insulin-like growth factor (IGF)-1, a key stimulator of protein synthesis, by the liver could affect muscle homeostasis in NAFLD [35]. A recent experimental study of diet-induced NAFLD in mice showed that low serum IGF-1 is associated with significant muscle atrophy and lower muscle strength, which is consistent with this hypothesis [36]. Second, the lower availability of amino acids, such as leucine and arginine, which are critical regulators of protein anabolism, and the resulting imbalance between muscle protein synthesis and degradation may contribute to poor muscle function in NAFLD [37]. Third, hepatic endoplasmic reticulum stress induced by steatosis can stimulate the secretion of hepatokines that may stimulate muscle breakdown [38]. In addition, muscle protein turnover can be influenced by various pro-catabolic molecules, such methylglyoxal or uric acid, that are released by the liver [37]. However, because most of these potential mechanisms are speculative, further studies are necessary to provide more information regarding the mechanisms of the muscle-liver interaction.

A literature search identified two publications regarding the link between HGS and NAFLD [39, 40]. However, the effects of sex and menopausal status in women were not adequately considered in these papers, and patients with critical diseases that affect muscle status, including stroke and renal failure, were not excluded or the effects adjusted for [39, 40]. In addition, the populations represented in these studies were very heterogenous, including both young and older people, and therefore a link between NAFLD with muscle strength in elderly individuals, who are most likely to have clinically significant sarcopenia, was not clearly identified. Therefore, the present study provides an advance, in that it shows that NAFLD might be a risk factor for muscle weakness during aging in both men and women, even when strict exclusion criteria are applied.

The major strength of this study is that it was a large population-based analysis, using fully representative nationwide data, which ensures its statistical reliability and power. In addition, low muscle strength was defined according to the Korea-specific HGS cut-off value, as recommended by the Working Groups for Sarcopenia [2, 11, 41]. However, despite these strengths, several limitations should also be considered when interpreting the results. First, hepatic imaging and histologic biopsy were not undertaken in the Korea National Health and Nutrition Examination Survey (KNHANES), mainly due to the high cost and associated risk, respectively. Instead, we adopted a risk model for the prediction of NAFLD. Although a high area under the curve (0.86) and high specificity (92%) have been demonstrated in Korea for HSI, which is a highly practical tool for such an assessment in a large general population cohort [42], it should be clearly stated that this model is not a confirmatory method for the diagnosis of NAFLD. Second, we are unable to infer causal relationships, due to the cross-sectional nature of this study. Third, our study population was exclusively Korean, and thus we cannot be certain that our results are applicable to other populations. Fourth, inevitable intra-individual variations in HGS measurements could have impacted our findings, although this is expected not to be a significant issue in this type of study employing a large sample size. Last, although we attempted to apply strict exclusion criteria and considered as many covariates as possible, the observed findings could have been due to uncontrolled comorbidities or medications that affect liver and/or muscle metabolism.

In conclusion, using a nationally representative sample of the Korean population, we have demonstrated that NAFLD is significantly associated with lower HGS and a higher risk of low muscle strength in both older men and women after adjustment for potential confounders. Considering that the maintenance of muscle and liver health and their interaction is of great interest in this era of aging populations, our study may establish a baseline for future well-designed prospective studies investigating the relationship between NAFLD and muscle strength, preferably using liver-imaging.

## METHODS

### Study population

This cross-sectional study used data acquired from the sixth edition of the KNHANES, which was conducted between January 2014 and December 2015. This nationwide survey used a stratified, multi-stage, clustered probability sampling method to select a representative sample of the non-institutionalized, civilian Korean population, as described previously [43–45]. Briefly, a three-stage sample design was used for the KNHANES. The primary sample units (PSUs) were selected from a sampling frame of all census blocks or resident registration addresses, and each PSU consisted of approximately 50–60 households. Following the selection of PSUs, all dwelling units in the PSU were listed and 20 households were selected in the field survey for household screening. The final stage of selection occurred in the household, in which all members aged ≥1 year were selected to participate. In this way, ~10,000 individuals were sampled in total across all 192 PSUs each year. The expected total sample size was based on past KNHANES iterations, using the response rates for each subdomain of interest. The goal for the overall response rate for the KNHANES was 75%. The survey provided extensive data regarding health and nutrition, which were collected at interview and through health examination, and the data were made publicly available at the KNHANES website (http://knhanes.cdc.go.kr; available in Korean and English).

In 2014 and 2015, the surveys were completed by 14,930 participants aged ≥1 year, and of these 13,387 participants were aged ≥10 years. HGS was measured in all the participants aged ≥10 years who gave their consent (*n* = 11,679), except for those who were excluded on the basis of the following criteria: absence of hands, arms, or thumbs, paralysis of the hands, presence of a cast on the hands or fingers, presence of a bandage on the hands or wrist, having undergone hand or wrist surgery in the preceding 3 months, or the presence of pain, tingling, or stiffness in the hands or wrists within the preceding week. Blood samples were also obtained from all participants aged ≥10 years who gave their consent (*n* = 11,378), except from those who had hemophilia, skin rash, open wound, weak blood vessels, vascular occlusion, paralysis, or a shunt for hemodialysis in both arms, or those who had received anticoagulant therapy or anti-cancer chemotherapy within the preceding month. Consequently, information regarding both HGS and blood biochemistry was available for 10,599 participants aged ≥10 years in 2014 and 2015. From this group, we enrolled 4,764 participants (2,323 men aged ≥50 years and 2,441 postmenopausal women) for the present study because we wished to focus on the risk factors for age-related loss of muscle strength.

Participants with neoplastic disease, sequelae of stroke, myocardial infarction, or renal failure were excluded from the study (*n* = 276) because these conditions could have affected muscle health. In addition, participants who met the following criteria were excluded on the basis of our protocol (*n* = 385): (1) excessive alcohol consumption, defined as ≥140 g alcohol/week for males and ≥100 g alcohol/week for females, or (2) positive serologic markers of hepatitis B or C viral infection. The remaining 4,103 participants (1,897 men aged ≥50 years and 2,206 postmenopausal women) were therefore eligible for this study. Because the KNHANES was a weighted survey, 4,103 participants were representative of the complete cohort of 22,596,690. All the participants in the KNHANES survey provided their informed consent. The KNHANES was reviewed and approved by the Ethics Committee of the Korea Centers for Disease Control and Prevention (KCDC).

### Measurement of HGS

HGS was measured using a digital grip strength dynamometer (T.K.K. 5401; Takei Scientific Instruments Co., Ltd., Tokyo, Japan), which has a measurement range of 5.0–100.0 kg force and an adjustable grip span. The minimum measurement unit was 0.1 kg. During the assessment, the participants were asked to stand upright with their feet hip-width apart and to look forward, with their elbow fully extended. The dynamometer was held by the test hand in a neutral, comfortable position (not flexed or extended), with 90° flexion of the index finger. The participants then performed three trials for each hand in an alternating fashion, starting with the dominant hand. They were instructed to squeeze the grip continuously, using full force, for at least 3 sec, and the interval between each trial was ~60 sec. Grip strength was recorded as the mean of three trials of the dominant hand [46]. In this study, the Korea-specific cut-off value of HGS was used to define low muscle strength, which was generated by deriving −2 standard deviation (SD) values for healthy young adults, as recommended by EWGSOP [11, 41]. The cut-off values for HGS were 28.9 and 16.8 kg in men and women, respectively [46, 47].

### Measurements of clinical and laboratory parameters

All participants underwent a thorough physical examination. Age, body weight, height, smoking habit, and resistance exercise performance (push-up, sit-up, dumbbell exercise, and horizontal bar) were recorded. Smoking and resistance exercise performance were grouped in three categories (never, past, or current; and no, intermittent [1–3 days/week], or regular [≥ 4 days/week], respectively). Height (cm) and weight (kg) were measured using standardized protocols while the participant was dressed in light clothing, without shoes. BMI (kg/m^2^) was calculated using height and weight. BP (mmHg) was recorded twice after resting for >15 min, using a mercury manometer and an appropriate size of cuff, and mean values were calculated.

Blood samples were collected from an antecubital vein in the morning following an overnight fast of ≥8 hours, immediately refrigerated, and then transported to the Central Testing Institute (Neodin Medical, Inc., Seoul, Korea), where they were analyzed within 24 h. Fasting serum total cholesterol and triglycerides were measured using enzymatic methods and a Hitachi automatic analyzer 7600 (Hitachi Ltd, Tokyo, Japan). Fasting glucose and HbA1c were measured using the hexokinase method on a Hitachi automatic analyzer 7600 (Hitachi Ltd.) and by high-performance liquid chromatography using an HLC-723G7 (Tosoh, Tokyo, Japan), respectively. Serum AST and ALT activities were measured using the IFCC method, without pyridoxal phosphate, and a Hitachi automatic analyzer 7600 (Hitachi Ltd). Serum hsCRP concentration was measured by immunoturbidimetry using a Cobas bio-centrifugal analyzer (Roche, Germany). Serum fasting insulin was measured by immunoradiometry using a 1470 Wizard gamma-counter (PerkinElmer, Turku, Finland). Insulin resistance was evaluated using the HOMA-IR index (HOMA-IR = [fasting serum glucose] × [fasting serum insulin]/405]) [48]. The intra- and inter-assay coefficients of variations in these analyses were consistently <3.5%.

### Definition of NAFLD

For the diagnosis of NAFLD, the HSI was calculated as follows: 8 × ALT/AST ratio + (BMI + 2 [if diabetic] + 2 [if female]). NAFLD was defined by an HSI value of ≥36. This prediction tool has been previously validated in >10,000 Korean subjects [49].

### Statistical analysis

Following the statistical guidelines of the Korean Centers for Disease Control and Prevention, all analyses were performed using the Complex Samples Plan, which is available as the Complex Samples option in SPSS Version 18.0 (SPSS Inc., Chicago, IL, USA). Survey sample weights were considered in all analyses to produce estimates that were representative of the non-institutionalized civilian population of Korea [50]. Data are presented as means with SDs or 95% confidence intervals (CIs), or as percentages, unless otherwise specified. The baseline characteristics were analyzed using unpaired *t*-tests for continuous variables and χ^2^-tests for categorical variables. To analyze the differences in HGS according to NAFLD status or HSI quartile, multivariable-adjusted least-square means with 95% CI were estimated and compared using analysis of covariance, after adjustment for confounders. The potential confounding factors included age, weight, systolic BP, smoking habit, resistance exercise category, serum total cholesterol, serum triglycerides, fasting HbA1c, and serum ALT, which were selected on the basis of their clinical applicability and/or statistical significance in univariate analyses. To generate ORs with 95% CIs for low muscle strength, according to the presence of NAFLD, per unit increase in HSI, or according to HSI quartile, we performed multiple logistic regression analyses after adjustment for confounding variables. To test the hypothesis that HSI might be independently associated with HGS, we performed multiple linear regression analyses using an Enter method, with HGS as a dependent variable and HSI as an independent variable. All the potential confounding factors (age, weight, systolic BP, smoking habit, resistance exercise category, serum total cholesterol, serum triglycerides, fasting HbA1c, and serum ALT) were also simultaneously considered in these analyses. *P* < 0.05 was considered to indicate statistical significance.

## Supplementary Material

Supplementary Figure 1

Supplementary Table 1
