# Supplementary material for: Lower hand grip strength in older adults with non-alcoholic fatty liver disease: a nationwide population-based study
Source: Aging (Albany NY). 2019 Jul 7;11(13):4547–60. doi: 10.18632/aging.102068 (PMC6660042; doi:10.18632/aging.102068)
Supplement: Supplementary Figure 1 [file aging-11-102068-s001.pdf]

SUPPLEMENTARY FIGURE

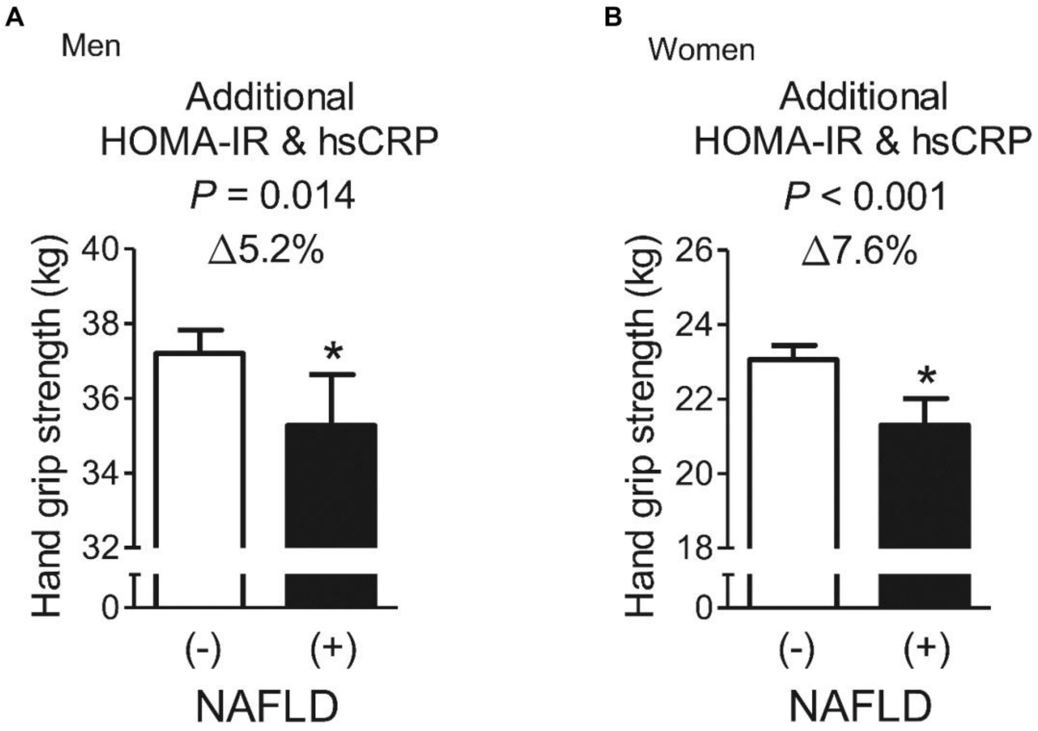

Supplementary Figure 1. Difference in hand grip strength between subjects with and without NAFLD after additional adjustment for fasting HOMR-IR and serum hsCRP in the multivariable model. (**A**) Men and (**B**) women. Values are presented as the estimated mean and 95% confidence interval, after adjustment for confounding factors. Delta (Δ) indicates a difference in hand grip strength from the control group. *Statistically significant difference from the control group. Multivariable model: adjustment for age, weight, systolic blood pressure, smoking habit, resistance exercise, total cholesterol, triglycerides, glycated hemoglobin A1c, and alanine aminotransferase. NAFLD, non-alcoholic fatty liver disease; HOMA-IR, homeostasis model assessment-estimated insulin resistance; hsCRP, high-sensitivity C-reactive protein.
