# Supplementary material for: Lower hand grip strength in older adults with non-alcoholic fatty liver disease: a nationwide population-based study
Source: Aging (Albany NY). 2019 Jul 7;11(13):4547–60. doi: 10.18632/aging.102068 (PMC6660042; doi:10.18632/aging.102068)
Supplement: Supplementary Table 1 [file aging-11-102068-s002.pdf]

SUPPLEMENTARY TABLE

Supplementary Table 1. Independent association between hepatic steatosis index and hand grip strength after additional adjustment for fasting HOMR-IR and serum hsCRP in the multivariable model.

Independent variables	Dependent variable: hand grip strength		
	**β**	**SE**	***P***
Men			
Age	**−0.322**	**0.024**	**<0.001**
Weight	**0.300**	**0.042**	**<0.001**
Systolic BP	0.005	0.015	0.723
Smoking habit	0.165	0.335	0.622
Resistance exercise	0.127	0.282	0.651
Serum total cholesterol	**0.014**	**0.007**	**0.035**
Serum triglycerides	−0.001	0.002	0.447
HbA1c	−0.331	0.299	0.270
Serum ALT	0.020	0.018	0.252
Serum hsCRP	−0.150	0.077	0.053
Fasting HOMA-IR	−0.104	0.101	0.303
Hepatic steatosis index	**−0.254**	**0.096**	**0.009**
Women			
Age	**−0.209**	**0.021**	**<0.001**
Weight	**0.226**	**0.029**	**<0.001**
Systolic BP	0.006	0.009	0.539
Smoking habit	−0.608	0.364	0.097
Resistance exercise	0.433	0.227	0.058
Serum total cholesterol	−0.004	0.004	0.333
Serum triglycerides	0.001	0.002	0.745
HbA1c	0.014	0.192	0.944
Serum ALT	**0.034**	**0.014**	**0.013**
Serum hsCRP	−0.072	0.080	0.370
Fasting HOMA-IR	**−0.094**	**0.040**	**0.021**
Hepatic steatosis index	**−0.304**	**0.067**	**<0.001**

The Enter method was applied to this model, with hand grip strength as a dependent variable, and with age, weight, systolic blood pressure, smoking habit, resistance exercise, total cholesterol, triglycerides, HbA1c, ALT, hsCRP, HOMA-IR, and hepatic steatosis index as independent variables, simultaneously. Values were adjusted for all the other variables in the table. **Bold** numbers indicate statistically significant values. β, regression coefficient; SE, standard error; BP, blood pressure; HbA1c, glycated hemoglobin A1c; ALT, alanine aminotransferase; hsCRP, high-sensitivity C-reactive protein; HOMA-IR, homeostasis model assessment-estimated insulin resistance.
